# The Rogue P-wave: Atrial Dissociation Following Bicaval Heart Transplantation

**DOI:** 10.19102/icrm.2019.100106

**Published:** 2019-01-15

**Authors:** William A. Huang, Jennifer P. Woo, Noel G. Boyle

**Affiliations:** ^1^UCLA Cardiac Arrhythmia Center, UCLA Health System, Los Angeles, CA, USA; ^2^Department of Medicine, David Geffen School of Medicine at UCLA, Los Angeles, CA, USA

**Keywords:** Atrial dissociation, bicaval, complete heart block, interatrial block, orthotopic heart transplant

## Abstract

We report the first case, to our knowledge, of atrial dissociation in a patient who underwent bicaval orthotopic heart transplantation. It was believed that atrioventricular dissociation prompting pacemaker implantation likely represented donor sinus bradycardia/arrest with intact atrioventricular conduction with surgically isolated recipient atrial activity.

## Introduction

Atrial dissociation consists of two P-waves, with one P-wave failing to conduct to the ventricles and being completely independent of the underlying rhythm.^1,2^ Atrial dissociation is a rare phenomenon and has been predominantly seen in critically ill patients with congestive heart failure and individuals undergoing orthotopic heart transplantation (OHT) via the biatrial technique.^1^ The biatrial technique anastomoses donor atria with recipient atria, which can result in a dual P-wave phenomenon due to coexisting donor and recipient sinus nodes that remain electrically isolated. Historically, the biatrial technique transitioned to the bicaval technique, beginning in approximately 2004, to overcome bradycardia and tachyarrhythmia complications from the biatrial technique. The bicaval technique preserves the right atrium and the anastomosis occurs in the superior vena cava (SVC) and inferior vena cava. As a result, the dual P-wave phenomenon is not expected.^3,4^ We present the first reported case, to the best of our knowledge, of dissociated P-waves in a patient who underwent bicaval OHT and required a pacemaker.

## Case presentation

**Figure 1:**
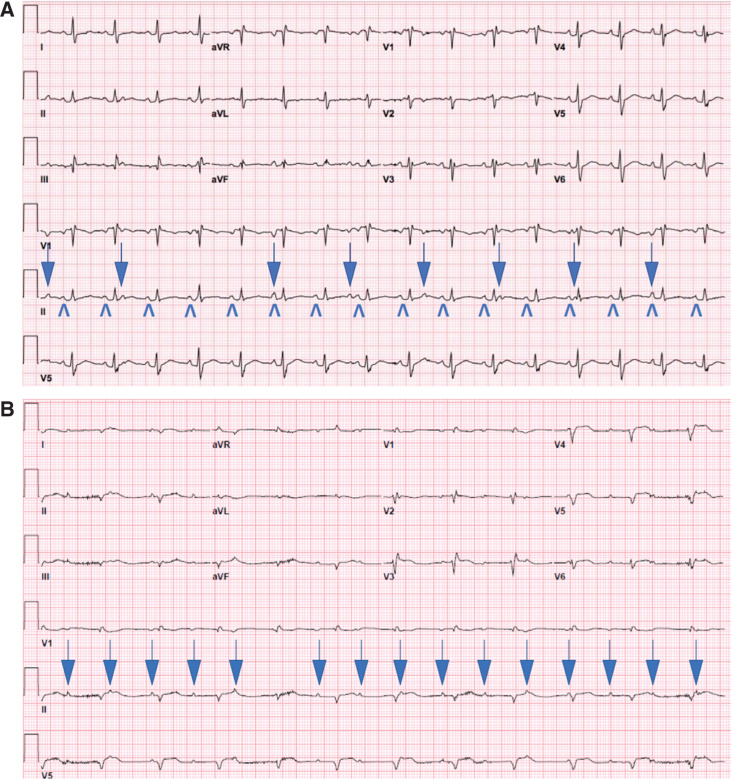
**A:** Recordings from the first postoperative day following OHT performed six years prior. Donor atrial rate was 97 bpm. Recipient atrial rate was 55 bpm (marked by ↓) and was dissociated from the donor atrial (marked by ∧) and ventricular rates. **B:** Presenting ECG. Complete heart block with junctional rate of 71 bpm and dissociated P-waves at a rate of 100 bpm. ST elevations are present in V3 to V6.

**Figure 2:**
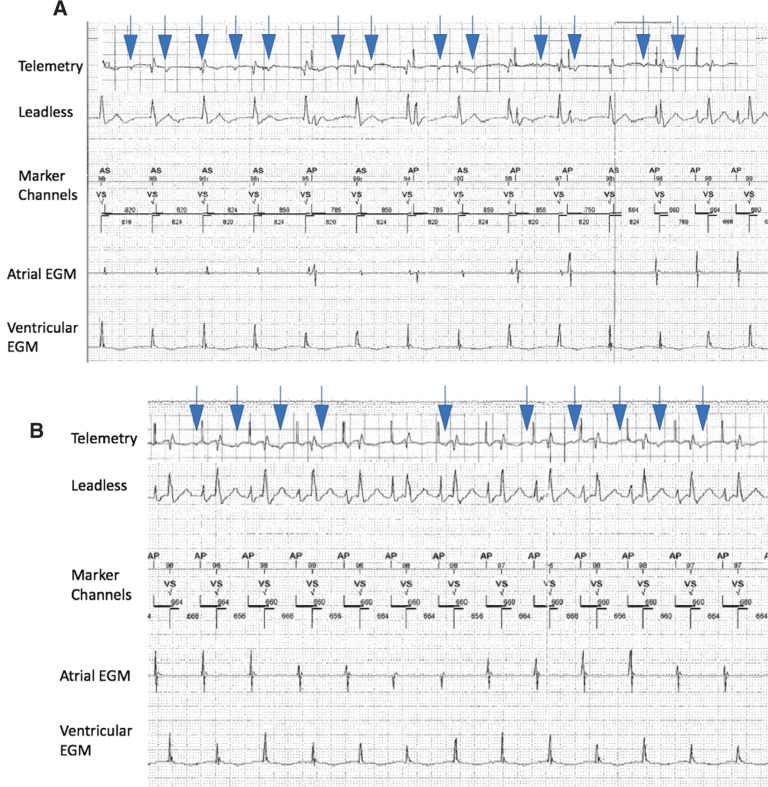
**A:** Pacemaker setting is AAI 70 bpm until the last three beats, at which point it transitions to AAI 90 bpm. Telemetry ECG lead V1 shows dissociated P-waves (marked by ↓) at a rate of 100 bpm to 110 bpm with an accelerated junctional rhythm of 73 bpm. Atrial electrograms, with the lowest sensitivity threshold, intermittently undersensed low-amplitude atrial signals with long AV intervals of 816 ms to 824 ms and, most notably, without evidence of sensing the dissociated surface P-waves. The last two beats of intracardiac tracing show that the small atrial signal is absent with atrial pacing and the acceleration of ventricular activity to 90 bpm with an AV delay of 200 ms, further supporting the idea that the small atrial signals represent donor atrium with isorhythmic dissociation from the underlying junctional rhythm. Alternatively, the low-amplitude atrial signals can represent far-field ventricular signals that are not present at the end of the strip due to amplitude autogain from the device programmer in the setting of consistent atrial pacing. **B:** Telemetry lead V1 shows P-waves (↓) at a rate of 100 bpm to 110 bpm with continued dissociation from atrial pacing at 90 bpm with 1:1 acceleration of the ventricle and an AV time of approximately 200 ms. The location of the atrial lead is therefore in the donor atrium and demonstrates intact antegrade AV conduction from this pacing site.

**Figure 3:**
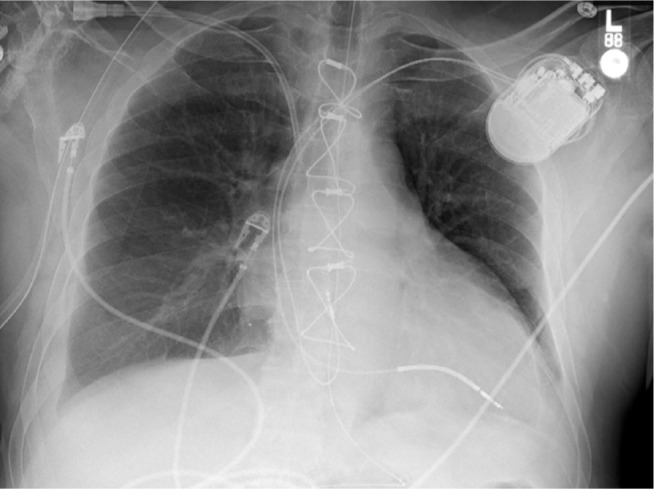
Portable chest X-ray demonstrates standard positions of the atrial lead and single-coil ventricular lead in the right ventricle.

A 62-year-old man who underwent bicaval OHT six years prior presented for medical attention with ST-elevation myocardial infarction requiring coronary stenting, cardiogenic shock requiring percutaneous left ventricular assist support, and atrioventricular (AV) dissociation. On review of prior electrocardiograms (ECGs) recorded immediately postoperatively following OHT **(Figure 1A)**, he showed unexpected evidence of dissociated P-waves representing dual atrial depolarizations despite a history of receiving OHT with the bicaval technique. Only one sinus node should be present, but the possibility of dual atrial tissues existing similar to in the case of the historical biatrial technique (donor and recipient) had to be entertained. The presenting ECG with AV dissociation **(Figure 1B)** could represent accelerated junctional rhythm in the setting of (1) sinus arrest of recipient atria and complete AV block of intact donor atrial P-waves or (2) sinus arrest of donor atria with accelerated junctional rhythm and surgically isolated recipient P-waves. Given the need for weaning inotropes safely, the decision was made to proceed with pacemaker implantation to treat either etiology. The atrial pacemaker lead showed small amplitude intermittent atrial signals that were slower and dissociated from the ventricular rate along with AV conduction times of more than 800 ms, which likely represented isorhythmic dissociation with underlying junctional rhythm. Atrial pacing at 90 bpm accelerated the ventricle in a 1:1 fashion with an AV conduction time of 200 ms, confirming that the donor atrium had normal AV conduction **(Figures 2A and 2B)**. As the P-waves continued to be dissociated despite atrial capture and ventricular acceleration, it was concluded that the dissociated P waves represented the electrically isolated recipient atrial activity. A chest X-ray demonstrated the standard atrial lead position **(Figure 3)**.

## Discussion

We present a unique case of atrial dissociation in a patient who underwent bicaval OHT. The patient’s second P-wave was thought to be likely resulting from residual atrial tissue from the recipient heart despite the patient having undergone a bicaval technique **(Figure 1A)**. Although uncommon, there have been reported cases of right atrial tissue extending as high as 4.7 cm above the sulcus terminalis in the SVC at postmortem pathology.^5^ Furthermore, these known variants of atrial extensions into the SVC have inherent automaticity and have been a focus of atrial tachyarrhythmia ablation.^6^ This patient’s SVC may have harbored extensions of recipient atrial tissue that were inadvertently preserved during his prior bicaval OHT operation and manifested as interatrial dissociation after his heart transplant. The possibility of pulmonary vein ectopy with surgical isolation accounting for the P-wave was considered but deemed unlikely given the negative V1 morphology of the P-wave. The postoperative dual P-wave baseline posed a diagnostic challenge to determine underlying rhythm when he presented with a single P-wave with AV dissociation; however, the possibilities of AV block versus donor sinus arrest warranted proceeding with a pacemaker.

Up to 25% of patients develop bradyarrhythmias after OHT, with a majority being sinus node dysfunction. Long-term outcomes demonstrate that the bicaval technique, as compared with the biatrial one, is less likely to require permanent pacemaker (PPM) implantation because the procedure preserves the integrity of the right atrium. In fact, the incidence of PPM implantation is as high as 9% in the biatrial group versus only 2% in the bicaval group.^7,8^ The mechanisms behind the high incidence of bradyarrhythmias varies but includes autonomic changes from surgical denervation, ischemic injury of the conduction system, and sinoatrial nodal artery abnormalities.^9^ This patient developed late-onset sinus bradycardia/arrest likely from a combination of ischemia and rejection in the setting of acute ST-elevation myocardial infarction and cardiogenic shock.

## Conclusion

To the best of our knowledge, this is the first reported case of atrial dissociation in a patient who underwent bicaval OHT. It was believed that AV dissociation necessitating pacemaker implantation likely represented donor sinus bradycardia/arrest with intact AV conduction with surgically isolated recipient atrial activity.

### Key teaching points

- Atrial dissociation following bicaval OHT is a rare phenomenon defined by two distinct and isolated P-waves.
- The use of the bicaval anastomotic technique can inadvertently result in biatrial anastomosis given the anatomic variability of right atrial tissue.
- AV dissociation can represent sinus arrest of donor atria with continuation of recipient atrial depolarizations or complete atrioventricular block. Both of these are indications for pacemaker implantation.
